# Mutations in the *Drosophila* homolog of human PLA2G6 give rise to age-dependent loss of psychomotor activity and neurodegeneration

**DOI:** 10.1038/s41598-018-21343-8

**Published:** 2018-02-13

**Authors:** Konstantin G. Iliadi, Oxana B. Gluscencova, Natalia Iliadi, Gabrielle L. Boulianne

**Affiliations:** 1 Program in Developmental and Stem Cell Biology, The Hospital for Sick Children, Toronto, Ontario M5G 1L7 Canada; 2 Department of Molecular Genetics, University of Toronto, Toronto, Ontario, M5S 1A8 Canada

## Abstract

Infantile neuroaxonal dystrophy (INAD) is a fatal neurodegenerative disorder that typically begins within the first few years of life and leads to progressive impairment of movement and cognition. Several years ago, it was shown that >80% of patients with INAD have mutations in the phospholipase gene, *PLA2G6*. Interestingly, mutations in *PLA2G6* are also causative in two other related neurodegenerative diseases, atypical neuroaxonal dystrophy and Dystonia-parkinsonism. While all three disorders give rise to similar defects in movement and cognition, some defects are unique to a specific disorder. At present, the cellular mechanisms underlying PLA2G6-associated neuropathology are poorly understood and there is no cure or treatment that can delay disease progression. Here, we show that loss of *iPLA2-VIA*, the *Drosophila* homolog of *PLA2G6*, gives rise to age-dependent defects in climbing and spontaneous locomotion. Moreover, using a newly developed assay, we show that *iPLA2-VIA* mutants also display impairments in fine-tune motor movements, motor coordination and psychomotor learning, which are distinct features of PLA2G6-associated disease in humans. Finally, we show that *iPLA2-VIA* mutants exhibit increased sensitivity to oxidative stress, progressive neurodegeneration and a severely reduced lifespan. Altogether, these data demonstrate that *Drosophila iPLA2-VIA* mutants provide a useful model to study human PLA2G6-associated neurodegeneration.

## Introduction

Phospholipase A2 (PLA2) belongs to a well-known superfamily of enzymes that hydrolyze the sn-2 ester of glycerophospholipids to produce a fatty acid and a lysophospholipid. This superfamily consists of more than 30 enzymes that can be divided into several groups based on their structures, catalytic properties and localizations^1,2^. Depending on the localization of the enzymes (during catalysis) they can be subdivided into two main categories: cytosolic and extracellular. In contrast to other, closely related cytosolic phospholipases A2, calcium- independent forms (iPLA2), do not require Ca^2+^ for activity. To date, the human iPLA2 family includes nine enzymes that exhibit either lipase or phospholipase activity. All members of this family share a patatin domain, which possesses lipid acyl hydrolase activity^3^. iPLA2-VIA (synonyms: GVIA PLA2, iPLA2-β, PNPLA9), encoded by *PLA2G6* gene, is probably the most studied enzyme of the iPLA2 family. The human *PLA2G6* gene gives rise to several splice variants^4^ that show different localization and probably cellular function^5,6^. The full-length cDNA encodes an 806-amino acid protein with a C-terminal lipase motif and 7–8 specific Ankyrin N-terminal repeats that may play a role in protein oligomerization and other protein-protein interactions^7^. There are also several conserved consensus sequences that include a putative ATP-binding domain, caspase-3 cleavage site and calmodulin-binding motif. The presence of these structural features suggests the functional importance of this enzyme in various biological processes. Indeed, in apoptotic cells, caspase-3 cleavage of iPLA2-VIA at one or several sites produces a truncated form of the enzyme that has enhanced catalytic activity^8^ and accelerates phospholipid turnover leading to apoptotic membranous changes^9^. iPLA2-VIA binds to calmodulin in a calcium- dependent manner^10^, playing an important role in the store-operated calcium influx pathway^11^. In recent years, several studies have demonstrated that iPLA2-VIA plays a key role in homeostatic phospholipid remodeling^12,13^, cell proliferation^14^, and regulation of immune cells^15^.

Interestingly, mutations in *PLA2G6* are associated with a variety of autosomal recessive neurodegenerative disorders with brain iron accumulation (NBIA Type 2). There are generally three overlapping PLAN (PLA2G6-associated neurodegeneration) phenotypes that can be distinguished based on age of onset and common pathological and behavioral features^16,17^. Early-onset of these disorders (classical infantile neuroaxonal dystrophy, INAD) is characterized by progressive motor and mental retardation, as well as truncal hypotonia and visual impairments. Atypical NAD demonstrates more phenotypic heterogeneity than INAD and disease onset may vary from early childhood to late teens. Patients with atypical NAD may show several clinical signs and symptoms similar to classical INAD, including gait instability and ataxia and often exhibit social communication, speech difficulties and autistic-like traits. The third phenotypic group is adult-onset dystonia-parkinsonism. Affected individuals initially display neuropsychiatric symptoms including depression, aggression, personality changes and rapid cognitive decline. Dystonia-parkinsonism manifests with resting tremor, generalized rigidity, bradykinesia and postural instability. Interestingly, brain iron accumulation, axonal swelling, spheroid bodies and cerebellar signs are typically observed in both infantile and atypical NAD while in dystonia-parkinsonism these pathological features are commonly absent^18^.

A number of animal models have been developed over the past decade to enhance our understanding of the neuropathology and genetics of PLAN. In 2008, the first PLAN mouse models were created by two independent groups. Both the iPLA2-VIA gene-targeted^19^ and knock-out^20^ mice developed age-dependent motor and sensorimotor impairments as well as widespread distribution of spheroids and vacuoles throughout the nervous system. In both studies, behavioral dysfunction and pathological features occurred in middle (about 13 months old) aged mice. However, in contrast to the phenotypes observed in null mutants, mice that produce a mutated protein (missense, non-conservative point mutation) exhibit early onset of disease that progresses quickly and more severely^21^. These mutant mice exhibit abnormal gait and poor performance in the hanging grip test around 7 weeks old. They also display widespread formation of spheroids containing tubulovesicular membranes and a recessive pattern of inheritance similar to human INAD. Interestingly, all of these mice show no phospholipase activity. Together, these results suggest that mutant forms of the iPLA2-VIA protein may contribute to early-onset of PLAN while the lack of protein plays an important role in age-dependent neuronal dysfunction, but not in the development of the nervous system.

In addition to the phenotypes described above, genetic ablation of PLA2G6 in mice also causes neuro-inflammation, significant loss of Purkinje cells and cerebellar atrophy^22^. Immunohistochemistry and ultrastructural analysis of spinal cords and sciatic nerves in *iPLA2-VIA* knock-out mice revealed that insufficient remodeling and degeneration of mitochondrial inner membranes and presynaptic membranes appear to be the factors most likely to be responsible for neuroaxonal dystrophy^23^. It also has been shown that both significantly reduced transcript levels of wild-type *iPLA2-VIA* and normal levels of expression of inactive mutant forms in mouse astrocytes severely disturb ATP-induced Ca^2+^ signaling^24^, suggesting a new mechanism that may be involved in the development of PLAN pathology.

Here, we have used two mutant *Drosophila iPLA2-VIA* alleles as well as transgenic RNAi approaches to investigate the consequences of total iPLA2-VIA deficiency or significant reduction of mRNA expression on lifespan, behavior, neurodegeneration and sensitivity to oxidative stress. We show that *iPLA2-VIA* mutant flies exhibit multiple phenotypes resembling those observed in PLAN patients. We show that both deletion of the entire *iPLA2-VIA* locus (genetic null mutant) and significant reductions in the levels of gene activity (P-element insertional hypomorphic mutant and RNAi knockdown), dramatically reduced the lifespan of middle-aged flies. Using a variety of approaches to measure locomotor behavior, we demonstrate that *iPLA2-VIA* mutant flies exhibit an age-dependent decrease in climbing ability and spontaneous locomotion. Moreover, using a recently developed method to examine psychomotor behavior in *Drosophila* we were able to detect impairments in fine-tune motor movements, motor coordination and psychomotor learning in the *IPLA2-VIA* mutant flies that are key features associated with PLAN disease. Finally, we show that mutations in *iPLA2-VIA* result in decreased oxidative stress resistance and severe age-related neurodegeneration in the brain.

## Results

### CG6718 is a fly ortholog of human calcium-independent phospholipase A2-VIA

**Figure 1:**
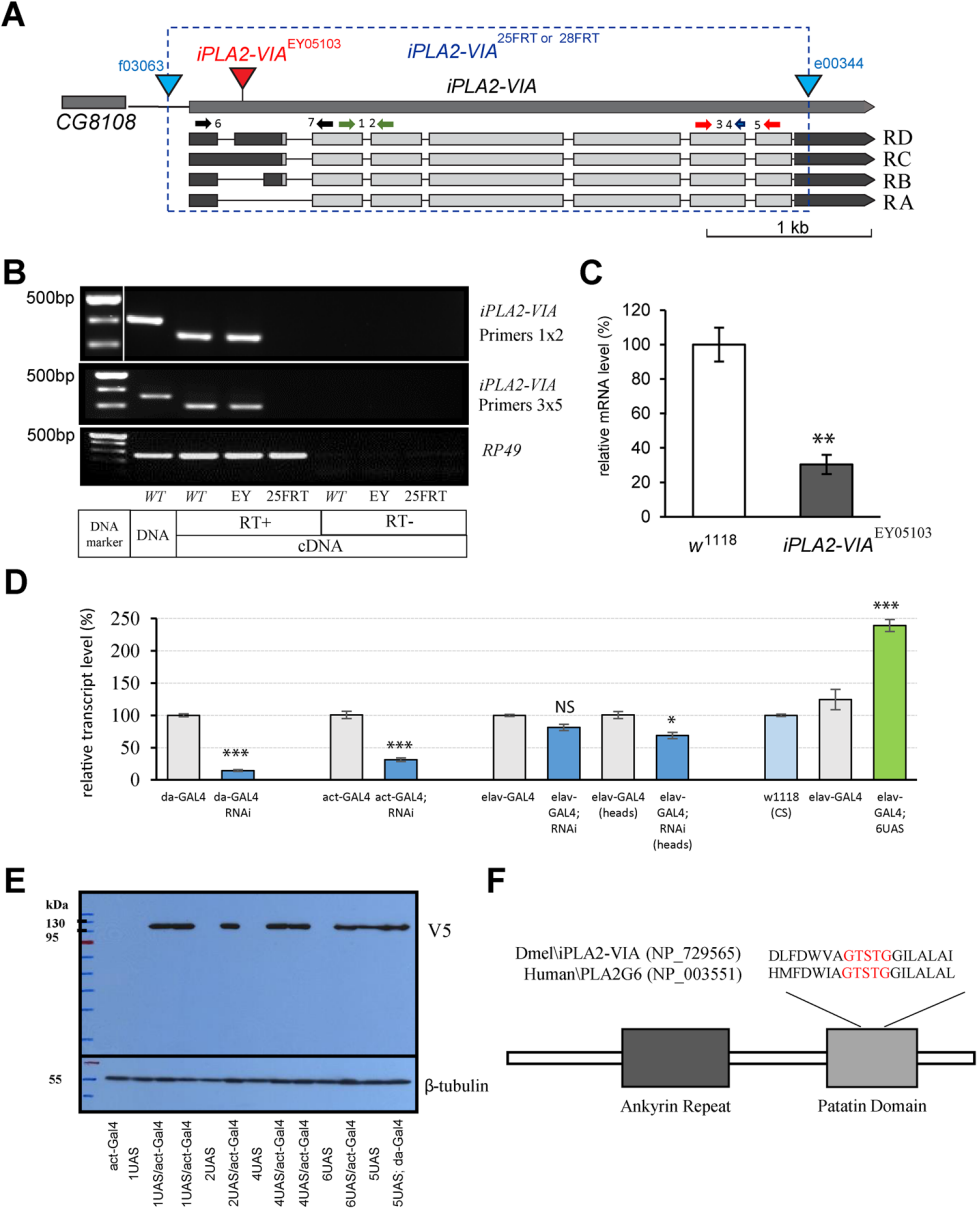
Characterization and expression of mutant alleles of *Drosophila iPLA2-VIA*. **(A**) Exon-intron organization of the *iPLA2-VIA* locus. Four transcripts RA, RB, RC and RD and the insertion site of the P element EPgy2-*iPLA2-VIA*^EY05103^ are shown. The dashed blue box indicates the region deleted by FLP/FRT mediated recombination, which lies between two insertions (blue triangles), and gave rise to two independent *iPLA2-VIA* alleles: *iPLA2-VIA*^25FRT^ and *iPLA2- VIA*^28FRT^. The following primer pairs, 1–2 and 3–4–5, were used for RT-PCR and qRT-PCR analysis. The 6–7 primer pairs were used in^25,26^. (**B**) A cropped gel image of the RT-PCR analysis of *iPLA2-VIA* alleles showing absence of expression in the null *iPLA2-VIA*^25FRT^ mutant and presence of the RT-PCR product in the *iPLA2-VIA*^EY05103^. Full-length gel images can be found in the supplementary materials (Figures S1 and S2). **(C)** qRT-PCR showing significant reduction in expression levels of *iPLA2-VIA* in the P-element mutant line. **(D)** Efficiency of ubiquitous (*da-GAL4* and *act-GAL4*) and pan-neuronal (*elav-GAL4*) RNAi-mediated knockdown and pan- neuronal overexpression of *iPLA2-VIA* transgenic lines. **(E)** A cropped gel image of the western blot probed with anti-V5 tag shows expression of five different UAS-*iPLA2-VIA* insertional lines. For all future experiments, we chose UAS-*iPLA2-VIA* line #6 (6UAS). Full-length gel images can be found in the supplementary materials (Figure S3). **(F)** Schematic representation of *Drosophila* iPLA2-VIA protein that contains an ankyrin repeat (N-terminal position) and a catalytic patatin domain. *Drosophila* iPLA2-VIA and Human PLA2G6 are aligned according to their highly conserved lipase consensus motif GXSXG. *, **, *** indicates P < 0.05, 0.01 and 0.001, respectively. Error bars represent standard error.

A homology BLAST-based search of the *Drosophila* database with human iPLA2-VIA amino-acid sequence (GenBank: CAG30429.1) revealed that the *Drosophila* genome contains a single copy of calcium-independent phospholipase A2-VIA gene, (*Drosophila* Computed Gene number: *CG6718*). The human and *Drosophila* proteins share 51% overall amino acid identity and 67% similarity with the highest degree of conservation in the patatin-like phospholipase domain, 57% and 72% respectively (NCBI-BLAST), which includes a serine lipase consensus sequence (GTSTG). Moreover, both human iPLA2-VIA and *Drosophila CG6718* display a similar N-terminal domain organization including multiple ankyrin repeats. According to the FlyBase annotation (http://flybase.org/reports/FBgn0036053.html) *CG6718* gives rise to 4 mRNA transcripts that encode 2 protein products (Fig. 1A). To determine the functional consequences associated with the loss of *Drosophila iPLA2-VIA*, we generated a null mutant allele (Fig. 1A,B, and supplementary materials Figure S1 and S2) by deleting the entire coding sequence of *CG6718* gene using a FLP-FRT mediated technique. Two independent lines, *iPLA2-VIA*^FRT25^ and *iPLA2-VIA*^FRT28^, were isolated and sequenced to confirm the presence of the deletion. Another allele of *iPLA2-VIA* used in this study was a P-element insertion mutant *iPLA2-VIA*^EY05103^ obtained from the Bloomington *Drosophila* Stock Center. This P- element line was previously characterized as a complete null by two groups of investigators^25,26^. However, our study shows that this is a hypomorphic mutant with significantly reduced mRNA expression rather than a null allele (Fig. 1C). The apparent discrepancy between our results may be due to the use of different primers for RT-PCR experiments. We designed two pairs of specific primers that covered different exons within the coding region (pairs 1–2 and 3–5, Fig. 1A), while they used primers around the P-element insertion site, which spans the first exon in the 5′UTR to the third exon in the coding sequence (pairs 6 and 7, Fig. 1A). Failure to detect any RT-PCR product in their experiments could be due to the presence of 5′ pre-mRNA that contains unremoved P-element sequence (there is about ten thousands of bps) or because imprecise removal of the P-element may produce a truncated 5′ form of mRNA.

To examine the effects of tissue-specific knockdown of *iPLA2-VIA* we used a UAS-*RNAi* line (HMS01544 - Harvard Medical School TRiP line collection) with no predicted off- target effects. Efficiency of knockdown was tested using *actin-5C*-*GAL4*, *daughterless*- *GAL4* (*da-GAL4*) and *elav*^c155^*-GAL4* (*elav-GAL4*) lines (Fig. 1D). Ubiquitous *iPLA2-VIA* knockdown displays the most dramatic ~60–80% reduction in whole body transcript levels compared to controls, whereas pan-neuronal knockdown, which could only be detected from isolated fly heads, significantly reduces the expression by approximately 30%. To examine the effects of *iPLA2-VIA* overexpression, we also generated a series of V5-tagged full-length *iPLA2-VIA* transgenic lines under the transcriptional control of the GAL4/UAS system. Western blot analysis against a V5 polyclonal antibody and real-time RT-PCR confirmed that the transgene was expressed and of the expected size for iPLA2-VIA-V5 (Fig. 1D,E and supplementary materials Figure S3).

### Loss of function mutations in iPLA2-VIA lead to shortened lifespan

**Figure 2:**
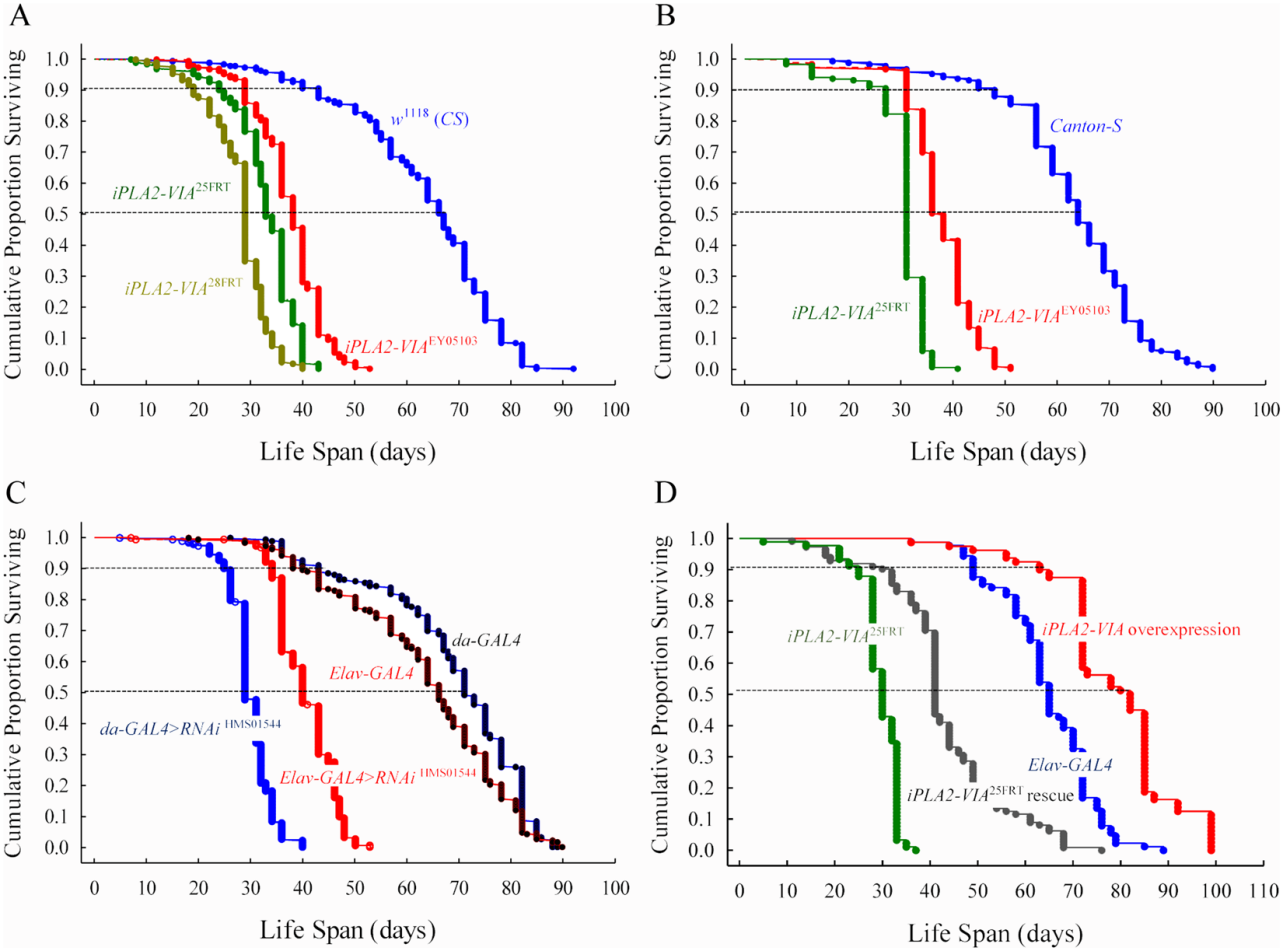
*iPLA2-VIA* mutants exhibit reduced lifespan. Kaplan–Meier survival curves for. (**A**) P-element mutant *iPLA2-VIA*^EY05103^ (n = 297, red color); null mutant lines: *iPLA2-VIA*^25FRT^ (n = 324, green color) and *iPLA2-VIA*^28FRT^ (n = 314, light green) and the genetic background, cantonized *w*^1118^ control line (n = 302, blue color). **(B)** Cantonized *w*+ ; *iPLA2-VIA*^25FRT^ null mutant line (n = 169, green color), cantonized *iPLA2-VIA*^EY05103^ (n = 213, red color) and wild-type *Canton-S* lines (n = 192, blue color). (**C**) Survival curves for ubiquitous da-*GAL4* > RNAi^HMS01544^ (n = 313, blue color) and pan-neuronal *elav-GAL4* > RNAi^HMS01544^ (n = 410, red color) RNAi-mediated knockdown and its control lines: *elav-GAL4* (n = 268, red color with black dots) and *da-GAL4* (n = 298, blue color with black dots). **(D)** Survival curves for *iPLA2-VIA*^25FRT^ null (n = 91, green color) and pan- neuronal rescue line *elav-GAL4*; *iPLA2-VIA*^25FRT^, 6UAS-*iPLA2-VIA* (n = 112, black color); *elav- GAL4* control line (n = 83, blue color) and *elav-GAL4* driven overexpression of *iPLA2-VIA* line (n = 80, red color).

In humans, mutations in *PLA2G6* are associated with severely reduced lifespan. To determine if loss of iPLA2-VIA also affects *Drosophila* lifespan we examined the survivorship curves for two independent *iPLA2-VIA* null mutant lines, a P-element insertional line and the genetic background control lines. Shown are the results generated by combining the data from two independent experiments (Fig. 2A). The log-rank test indicated highly significant differences in lifespan between control and mutant lines (p < 0.0001). The mutant lines show about 50% decrease in median lifespan. These results are consistent with that obtained from another experiment where the mutant lines were first converted to a *Canton S* wild type background (Fig. 2B). Interestingly, in both sets of experiments, we observed a dramatic effect on survival in mutant *iPLA2-VIA* flies during a distinct time interval corresponding to middle age (e.g. four-week old flies).

To further demonstrate an association between *iPLA2-VIA* and lifespan, we used a combination of RNAi lines to knockdown expression and transgenic lines to examine the effects of overexpression. We found that RNA-mediated gene silencing using either a pan- neuronal driver (*elav*^c155^*-GAL4)* or ubiquitous driver (*da-GAL4)* to knockdown of *iPLA2- VIA*, significantly reduced lifespan in a similar manner to that observed in null and P- element mutants (Fig. 2C). Moreover, there is a remarkable analogy in the shape of survivorship curves and median lifespan between flies with targeted pan-neuronal knockdown of *iPLA2-VIA* and the original P-element mutant (median lifespan: *elav*^*c155*^*- GAL4* > *RNAi = *40 days; *iPLA2-VIA*^EY05103^ in a *w*^1118^ *CS* background = 38 days; *iPLA2-*_*VIA*_^EY05103^ in a CS background = 38 days) as well as between *ubiquitous da-GAL4* knockdown and null mutant lines (median lifespan: *da-GAL4* > *RNAi* = 29 days; *iPLA2- VIA*^FRT25^ in *w*^1118^ *CS* background = 33 days; *CS* background = 31 days and *iPLA2-VIA*^FRT28^ in a *w*^1118^ *CS* background = 29 days). Importantly, crossing UAS-*iPLA2-VIA* transgenic flies to an *elav*^*c155*^*-GAL4* driver led to a partial rescue of the null mutant phenotype and significantly (p < 0.001) extended lifespan in the *iPLA2-VIA* wild type background (Fig. 2D). It is noteworthy that the shape of the lifespan curves of the null mutant and *elav*^*c155*^*-GAL4* control line are comparable with those derived from previous experiments (Fig. 2A–C) while the rescue and overexpression curves exhibit some aberrant patterns. The rescue line initially shows a prolonged shoulder compared to the null mutant followed by a sharp drop in survival, which is about 10 days later than that observed in the null mutant.

Remarkably, although the rescued lines still exhibit a steep drop in the survival curve, this is followed by a long tail that extends out for a longer time than what we observe in the *elav*^*c155*^*-GAL4* control. The survivorship curve of flies overexpressing *iPLA2-VIA* also exhibit an extended plateau lasting for about 70 days followed again by a steep drop-off that is separated by two intermediate shoulders with a small number of deaths. Overall, pan- neuronal overexpression of *iPLA2-VIA* extends the median lifespan of null mutants and wild type flies by 11 and 17 days respectively.

### Lack of the iPLA2-VIA causes progressive age-dependent motor dysfunction

Since loss of motor skills is one of the major symptoms of PLAN, we examined different aspects of motor behavior using a variety of techniques. As described above, the lifespan of iPLA2-VIA knockdown and mutant flies is about twice as short compared to their respective genetic background controls. Therefore, to distinguish between impairments that are due to physiological rather than chronological aging, motor behaviors were measured at several chronological time-points. Chronological age refers to how old flies actually are and can be defined as the number of days after eclosion. In contrast, physiological age is defined as the level of cumulative survivorship. If the primary effect of mutations in iPLA2-VIA is to shorten lifespan, then we should not observe any differences in behavior when we compare mutants with controls at the same physiological age. Based on the survivorship curves, all locomotor-associated behaviors were assessed in young flies (2 weeks old), when the physiological age for mutants and the control line was similar; right before mutant flies entered the rapid death stage (4 weeks old; ~90% of survival) and in control flies at that is the same physiological age (6 weeks old; ~90% of survival).

**Figure 3:**
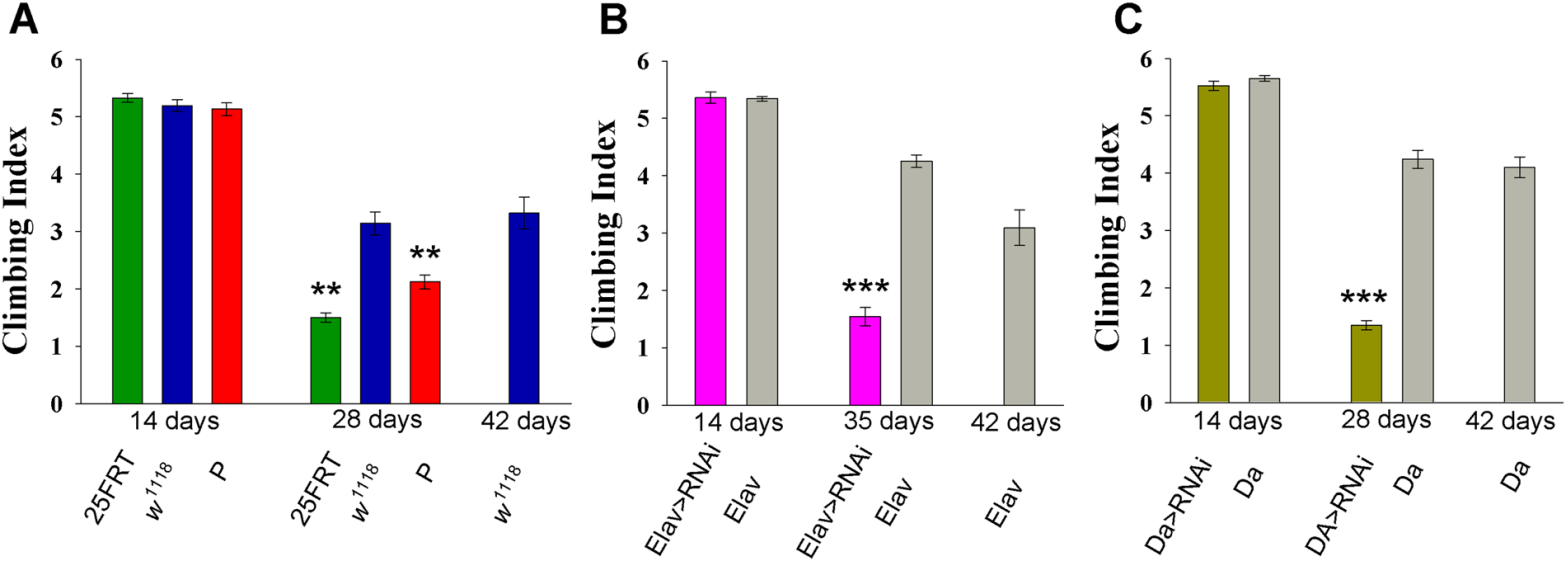
*iPLA2-VIA* mutants display impaired climbing ability. **(A)** Climbing index derived from *iPLA2-VIA*^25FRT^ null (green color), P element insertional *iPLA2-VIA*^EY05103^ (red color) line and cantonized *w*^1118^ control line (blue color) at different age groups. **(B)** Age-dependent reduction in climbing ability observed after pan-neuronal (*elav-GAL4*) and **(C)** ubiquitous (*da- GAL4*) knockdown of *iPLA2-VIA*. N is at least 8 (trials) per genotype and time point. **, *** indicates P < 0.01 and 0.001, respectively. Error bars represent standard error.

**Figure 4:**
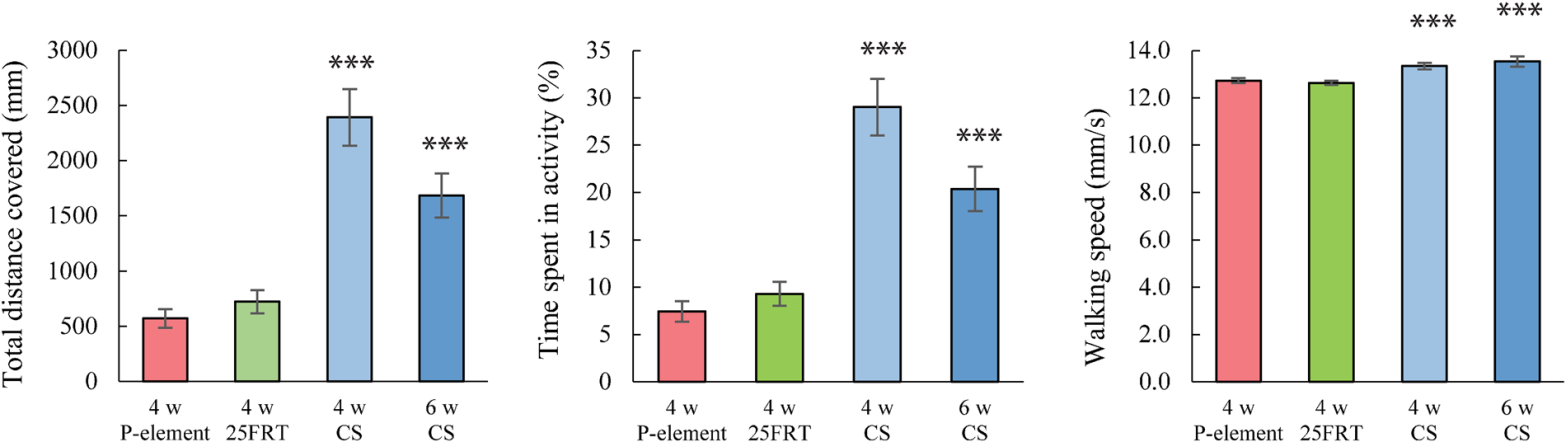
*iPLA2-VIA* mutants display impaired locomotor activity. Open-field 10 min video- tracking data for four-week old *iPLA2-VIA*^25FRT^ null (green color), P element insertional *iPLA2- VIA*^EY05103^ (red color) line and four (light blue color) and six-week old *Canton S* control line (blue color). (**A**) The total distance covered in 10 minutes (mm); **(B**) Percent activity over time; **(C)** Mean of walking speed (mm/s). N is at least 34 animals per genotype and time point. *** indicates P < 0.001. Error bars represent standard error.

We first tested the motor function using a countercurrent apparatus. This apparatus has several advantages, which make it ideal for initial analysis of motor-related behaviors. Specifically, the countercurrent apparatus integrates many activities including negative geotaxis response, climbing ability, escape response and locomotor activity itself, thereby making it possible to detect and quantify putative deficits in any of these motor activities. There were no significant differences in the Climbing Index (CI) between two-week old control *w*^1118^ (*CS*) and *iPLA2-VIA* mutant genotypes (Fig. 3A; ANOVA: *F*_(2, 21)_ = 1.026, *p* = 0.376). Unlike young flies, both four-week old P-element and null mutant flies showed a significant decrease in CI compared to either chronologically (four-week old) or physiologically (six-week old) matched controls (ANOVA: *F*_(3, 35)_ = 30.111, *p* < 0.001) (Fig. 3A). To confirm that the progressive locomotor deficit observed in *iPLA2-VIA* mutants was due to loss of *iPLA2-VIA* activity we knocked down *iPLA2-VIA* pan-neuronally and ubiquitously using *elav-GAL4* and *da-GAL4* drivers respectively. Based on the cumulative survivorship data (Fig. 2C) we performed experiments in young (2 weeks old) flies (when all genotypes show similar chronological and physiological age) (Fig. 3B,C). At this age, both knocked down lines show no differences from their controls. However, 5-week old *elav- GAL* > *RNAi* (Fig. 3B) and 4-week old *da-GAL4* > *RNAi* (Fig. 3C) flies exhibit significantly reduced climbing ability compared to either chronologically or physiologically matched (6 weeks old, ~90% of survival for all variants) controls. These data clearly suggest a strong *iPLA2-VIA* neuronal contribution to the observed behavioral phenotype.

To further dissect the cause of the climbing deficits observed in four-week old *iPLA2-VIA* mutant flies we performed a comprehensive analysis of spontaneous locomotor activity using an open field assay combined with a video-tracking system. Both mutant lines exhibited a significant decrease in all measured parameters of locomotor activity, including time spent in locomotor activity, average walking speed and total distance covered compared to their chronological and physiological controls (Fig. 4). Of these parameters, the walking speed is more directly associated with physical activity status. Remarkably, although mutant flies display significantly reduced walking speed, the most prominent differences (almost four-fold) between control and both mutant lines were found in time spent in locomotor activity and total distance covered. These data are coincident with our earlier observations and may suggest that four-week old mutant flies are less active overall and their physical ability to walk in the open field is less affected.

**Figure 5:**
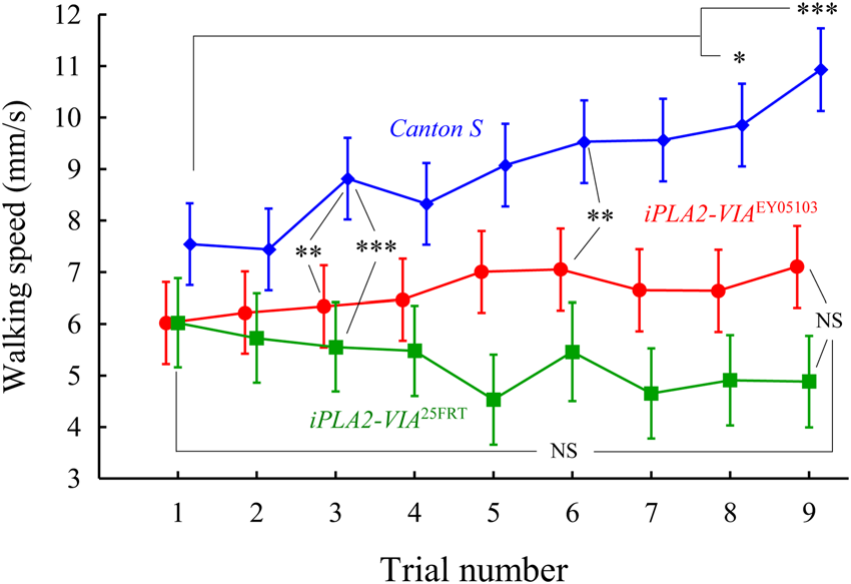
*iPLA2-VIA* mutants exhibit reduced walking speed. Mean walking speed for *Canton S* wild type (blue color curve) and *iPLA2-VIA*^25FRT^ null (green color) and P element insertional *iPLA2-VIA*^EY05103^ (red color) flies plotted as a function of the number of successive trials. Tukey HSD post-hoc comparisons show differences between mutant and control lines in the mean of walking speed. Vertical bars denote 0.95 confidence intervals. Also, shown are the comparisons between first and last trial within the same genotype. * denotes significant differences at *p* < 0.05, ** *p* < 0.01 or *** *p* < 0.001; NS denotes non-significant.

In our previous studies, we showed that two-week old *iPLA2-VIA*^EY05103^ P-element mutant flies exhibit similar locomotor parameters to that of control flies whereas their fine-motor movements, motor coordination and psychomotor learning were significantly impaired^27^. Our attempts to measure psychomotor behavior in four-week old P-element mutant flies were unsuccessful due to their inability to walk along a tensioned wire between the tips of two platforms submerged in water. Thus, in this study, we attempted to investigate the performance in the “equilibrist test” using three-week old control and mutant flies. Also, to validate previous results obtained from the P-element line we included in the assay a second (null) mutant line. Since body size/width of individual flies may have an impact on performance in the equilibrist test, we first measured width of control and mutant males. The width of a male thorax was measured as the distance between the posterior sternopleural bristles on the ventral surface of the thorax^28^. Fly images were captured using the DEM200 digital camera attached to a Nikon SMZ645 stereoscope and then analyzed with micro- measure 1.20 software. There were no statistically significant differences between genotypes as determined by one-way ANOVA: *F*_(2, 62)_ = 0.234, *p* = 0.792 (*Canton S* = 0.619 mm ± 0.04 (n = 22); *iPLA2-VIA*^EY05103^ = 0.617 mm ± 0.04 (n = 23); *iPLA2-VIA*^FRT25^ = 0.614 mm ± 0.05 (n = 20); Levene’s Test for Homogeneity of Variances: *F*_(2, 62)_ = 0.721, *p* = 0.490). Two-way ANOVA of walking speed detected a significant main effect of genotype: *F*_(2, 1123)_ = 174.30, *p* < 0.001 and trails *F*_(8, 1123)_ = 2.8401, *p* = 0.004. Post-hoc comparisons showed that both P- element and null mutant flies were unable to improve their performance in the “equilibrist test” (Fig. 5). The mutant flies showed no significant difference in walking speed between the first (1) and last (9) trials (Fig. 5). In contrast to *iPLA2-VIA* mutants, control flies displayed a considerable trend toward significance between the first and following trials, which became statistically significant after 7 trials. Furthermore, control *Canton S* flies demonstrated a significantly higher walking speed that could be detected as early as the third trial compared to null mutants and after the fifth trial compared to P-element mutant flies. Altogether, these experiments revealed significant age-depended impairments in *iPLA2-VIA* mutant flies in locomotor activity and psychomotor learning.

### Mutations in the Drosophila iPLA2-VIA do not affect olfactory associative learning and memory

**Figure 6:**
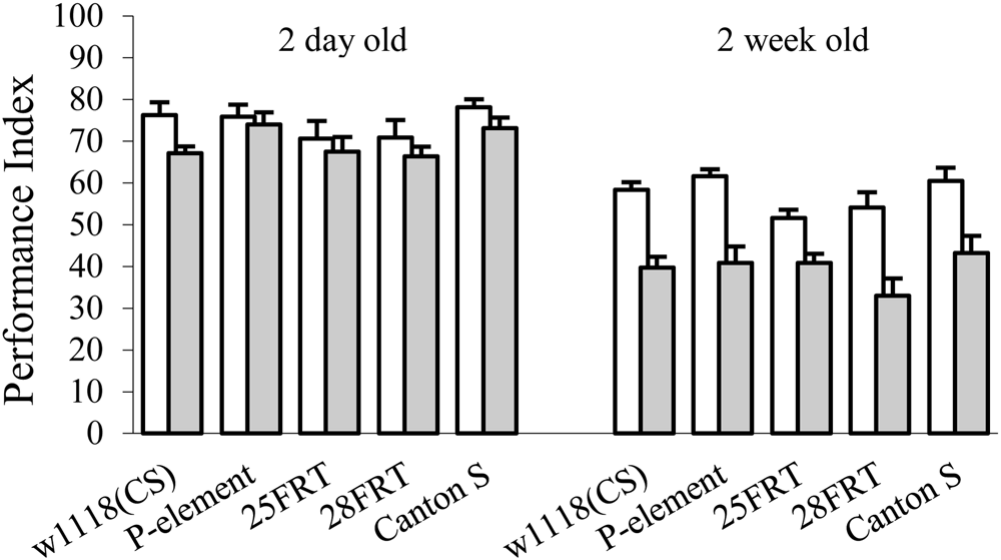
Olfactory associative learning and memory in *iPLA2-VIA* mutant. Memory retention was tested immediately (non-shaded column) or 30 min (shaded column) after training. There were no differences between *iPLA2-VIA* mutant and control lines neither between young 2 day old nor between two week old flies. N is at least 6 PIs per time point/variant. Error bars represent standard error.

Adult-onset of PLA2G6-associated neurodegeneration is frequently accompanied by progressive and striking psychiatric deficits and cognitive decline. To study potential cognitive deficits associated with mutations in *iPLA2-VIA*, we examined Pavlovian olfactory associative learning and memory in adult flies. To ensure that sensorimotor activities necessary for olfactory associative learning in mutant flies were indistinguishable from that of controls we first tested their ability to avoid electric shock and odors. The results of these tests are summarized in Table 1 and demonstrate that mutant flies show normal sensorimotor responses to the task-relevant stimuli. One-way ANOVA also did not identify any significant differences between genotypes in learning: *F*_(4,27)_ = 1.013, *p* = 0.418 or short- term memory performance: *F*_(4, 25)_ = 1.978, *p* = 0.129 in two-days old flies (Fig. 6). To explore the possibility that *iPLA2-VIA* mutants may exhibit age-dependent defects in learning and memory we used the same assay to test two-week old flies. We found no significant differences between any of the genotypes in learning *F*_(4,26)_ = 2.392, *p* = 0.077 and short-term memory *F*_(4, 25)_ = 1.208, *p* = 0.033 (Fig. 6). These experiments show that lack of *iPLA2-VIA* does not affect *Drosophila* olfactory associative learning and memory in 2–3 day-old and two-week old flies and has no impact on task-relevant sensorimotor responses.

**Table 1:** Olfactory Avoidance and Shock Reactivity.

Genotype	N	3-OCT Acuity	MCH Acuity	Shock Avoidance
*Canton S*	6	56.55 ± 2.75	53.11 ± 4.33	68.88 ± 5.22
*w*^1118^ (*CS*)	6	59.80 ± 3.71	57.59 ± 5.20	72.55 ± 4.74
25FRT	6	60.73 ± 3.63	58.08 ± 3.69	73.98 ± 3.46
28 FRT	6	67.09 ± 2.86	67.30 ± 4.84	64.36 ± 6.63
*P-element*	6	64.62 ± 2.13	65.19 ± 3.32	61.97 ± 5.85

Avoidance and shock reactivity scores are indistinguishable by overall ANOVA (3- OCT, *p* = 0.16; MCH, *p* = 0.16; shock avoidance, *p* = 0.45). 25FRT and 28FRT are *iPLA2-VIA*^FRT25^ and *iPLA2- VIA*^FRT28^ respectively. P-element is *iPLA2-VIA*^EY05103^.

### ***Drosophila*** calcium-independent phospholipase A2-VIA deficiency results in progressive neurodegeneration

**Figure 7:**
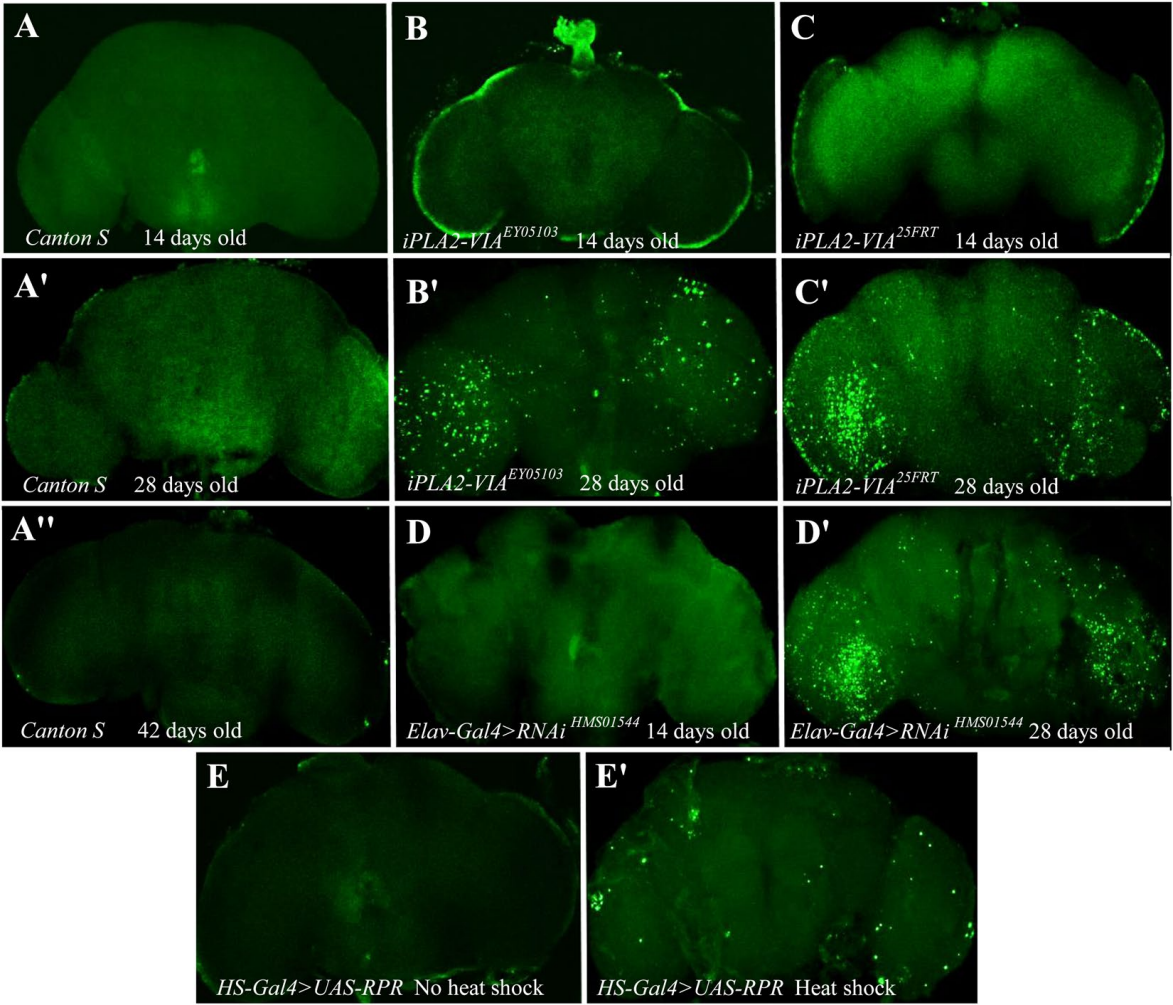
*iPLA2-VIA* mutants show age-dependent neurodegeneration revealed by TUNEL staining. Confocal microscopy images of adult brains are shown. *Canton S* **(A,A’ and A”)** and UAS-rpr **(E** and **E’)** were used as negative and positive controls, respectively. *UAS-rpr* line was crossed with *hs-GAL4* and raised at 18 °C. Their F_1_ progeny were also raised at 18 °C and 5–7 days after eclosion flies were heat-shocked at 38 °C for 20 min. 5 days after heat shock brains of heat-shocked **(E’)** and non-heat-shocked control flies **(E)** were analyzed. No neurodegeneration was found in the brains of 14 day old mutant (**B** and **C)** and pan-neuronal knockdown flies **(D)**. Brains dissected from both 28 day old *iPLA2-VIA*^25FRT^ null and P element insertional *iPLA2- VIA*^EY05103^ mutants as well as from 28 day old *elav-GAL4* > *RNAi*^HMS01544^ knockdown flies **(D’)** show a very prominent number of apoptotic positive cells compared to physiologically and age- matched wild type *Canton S* controls **(A,A’ and A”)**.

Shortened lifespan and age-dependent progressive motor dysfunctions are often associated with neurodegenerative pathologies^29^. Recently, morphological examination of the fly eye and thin paraffin sections of *iPLA2-VIA*^EY05103^ P-element mutant fly brains revealed abnormal retinal structures and widespread vacuolation in 32-day-old flies^26^. These pathological features could reflect an increase in the number of dead cells. We therefore studied apoptotic cell death by TUNEL staining in whole-mount fly brains. As a positive control, apoptosis in adult flies was induced by misexpression of *UAS-Reaper*^30^ driven by a heat shock-inducible GAL4 (Fig. 7E,E’). In *Drosophila*, activation of p53 in response to DNA damage initiates synthesis of Reaper, which in turn binds to DIAP (*Drosophila* Inhibitor of Apoptosis Protein), inactivating its ability to bind and inhibit caspases, leading to apoptosis^31^. DNA cleavage in apoptotic cells can be detected by terminal deoxynucleotidyl transferase (TdT)-mediated dUTP nick end labeling (TUNEL). We found no TUNEL-positive cells in the brains of either two-week old *iPLA2-VIA* mutants or wild- type *CS* flies (Fig. 7A–C). In contrast, by four weeks of age, the number of TUNEL-positive brain cells was dramatically higher in both P-element and null mutant flies (Fig. 7B’,C’) while no TUNEL-positive cells were detected in the brains of physiologically (6-week old) and chronologically (4-week old) matched controls (Fig. 7A’,A”). Strikingly, knockdown of *iPLA2-VIA* using the pan-neuronal driver, *elav*^*c155*^*-GAL4*, results in severe neurodegeneration in the brains of 4-week old flies, while 2-week old flies do not show a positive TUNEL staining (Fig. 7,D,D’).

### Impact of induced oxidative stress on survival of iPLA2-VIA mutant flies

**Figure 8:**
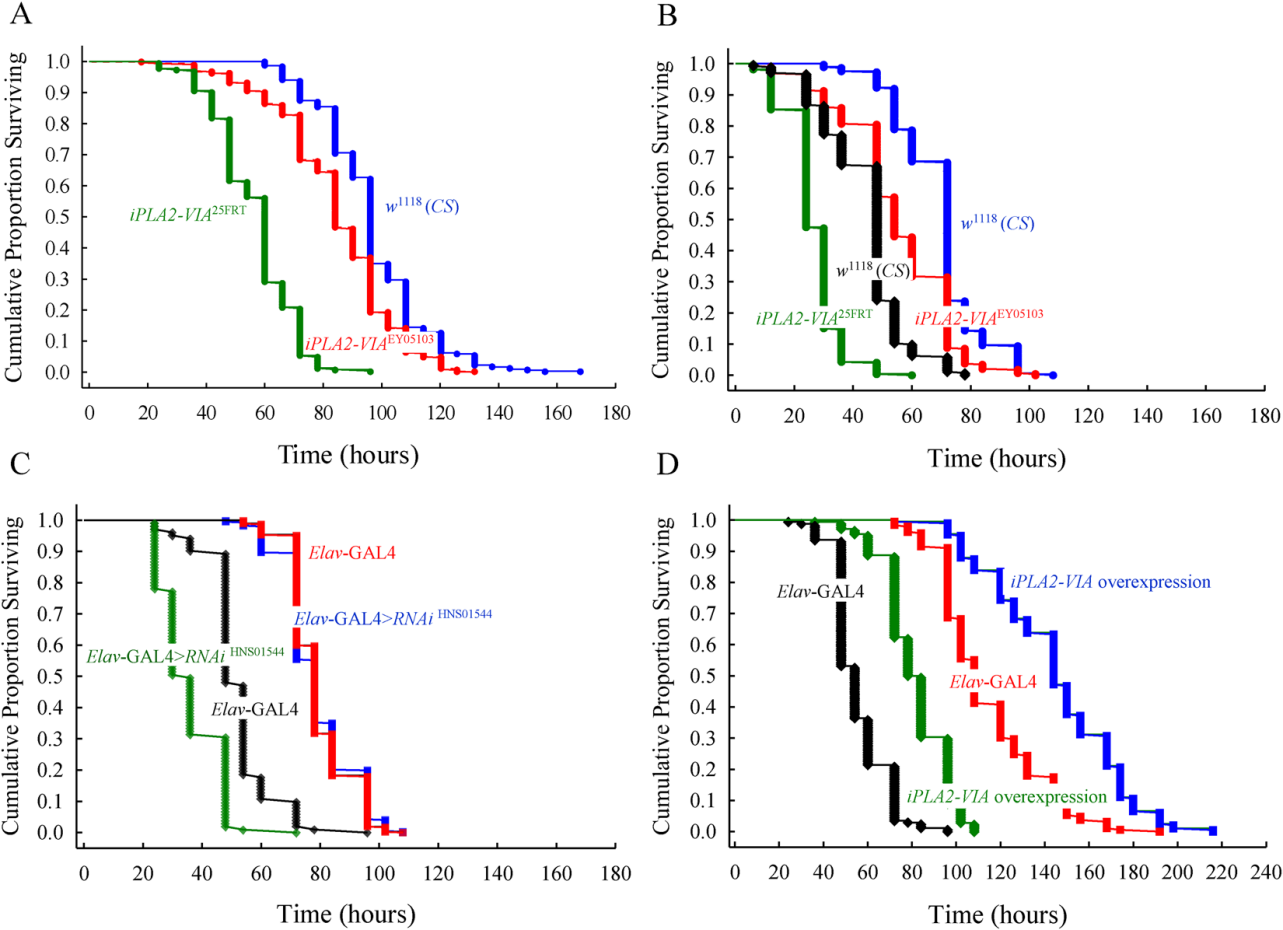
Resistance to 2% H_2_O_2_ induced oxidative stress in *iPLA2-VIA* mutant flies. **(A**) Survival curves for 7 day old P element mutant *iPLA2-VIA*^EY05103^ (n = 299, red color); null mutant lines: *iPLA2-VIA*^25FRT^ (n = 224, green color) and its genetic background cantonized *w*^1118^ control line (n = 303, blue color). **(B)** Survival curves for 28 day old P element mutant *iPLA2-VIA*^EY05103^ (n = 296, red color); null mutant lines: *iPLA2-VIA*^25FRT^ (n = 280, green color) and for age-matched (n = 300, blue color) and physiologically matched 42 day old cantonized *w*^1118^ control line (n = 274, black color). **(C)** Effect of pan-neuronal *elav-GAL4* > RNAi^HMS01544^ (n = 362, blue square) knockdown and *elav-GAL4* control (n = 397, red square) lines on survival of 7 day old flies and on survival of 28 old *elav-GAL4* > RNAi^HMS01544^ (n = 105, green diamond) knockdown and *elav-GAL4* control (n = 102, black diamond) lines. **(D)** Effect of *elav-GAL4* driven overexpression of *iPLA2- VIA* (n = 199, blue square) and *elav-GAL4* control (n = 189, red square) on H_2_O_2_ oxidative stress resistance for 7 day old and for 42 old *iPLA2-VIA* overexpression (n = 178, green diamond) and *elav-GAL4* control (n = 173, black diamond) lines.

To determine whether the high level of apoptosis observed in the brains of mid-aged *iPLA2- VIA* mutants can be attributed to oxidative stress, we exposed control, mutant and transgenic flies to hydrogen peroxide. Both young and mid-aged *iPLA2-VIA*^EY05103^ mutant flies showed significantly reduced survival rates (P < 0.001) in the presence of H_2_O_2_. The median lethal time (LT50) in the P-element mutant line was decreased by 12.5% and 25% for young and mid-aged flies, respectively, compared to the genetic background control *w*^1118 ^*CS* line (Fig. 8). Similar treatment of *iPLA2-VIA*^FRT25^ null mutant flies resulted in an even more dramatic reduction in oxidative stress resistance compared to the P-element mutant: LT50 decreased by 28.6% for young and by 55.5% for mid-aged flies or to controls: LT50 decreased by 37.5% for young and by 67% for mid-aged flies. On the other hand, comparisons made at the same physiological age between mid-aged flies (4 weeks of age for mutant and 6 weeks for control) show that control *w*^1118^*CS* flies exhibit an LT50 that is approximately 50% higher than the null mutant. However, they are less resistant to oxidative stress than P- element mutant flies. To further demonstrate a role of *iPLA2-VIA* in resistance to H_2_O_2_- induced oxidative stress, we examined the effect of knockdown or overexpression using a pan-neuronal driver. Interestingly, *RNAi-*mediated knockdown of *iPLA2-VIA* did not affect sensitivity to oxidative stress in 1-week old flies (*p* = 0.80), whereas 4-week old knockdown flies were significantly more sensitive to administration of H_2_O_2_ (*p* < 0.001) (Fig. 8C). Constitutive pan-neuronal overexpression of *iPLA2-VIA* under control of *elav*^c155^*-GAL4* has a positive effect on both 7 and 42 day-old flies significantly increasing their survival under oxidative stress (Fig. 8D).

## Discussion

Classical forms of infantile neuroaxonal dystrophy (INAD) were first described in the early 1950s by Franz Seitelberger and until recently, were diagnosed based on clinical and pathological findings. However, since the discovery that almost all cases of INAD are associated with mutations in *PLA2G6*, genetic testing is used to confirm the diagnosis. To date, PLA2G6-associated neurodegeneration (PLAN) is classified into three types based on age of onset: infantile-onset (classic INAD), childhood-onset (atypical NAD) and adult- onset (dystonia-parkinsonism). PLAN is a rare autosomal recessive disorder. The *PLA2G6* mutation detection rate varies from 90% in cases with classic NAD phenotype to 100% for dystonia-parkinsonism^18^. Since the phenotypic presentation of atypical NAD is quite varied, and most of the described data were obtained before the availability of genetic analysis, the detection rate for this type of disease remains unclear. So far 129 mutations in *PLA2G6* have been reported in patients with PLAN^32^ including missense, nonsense and splice site mutations, small exonic deletions and duplication. The vast majority of mutations were found in the coding region of *PLA2G6*. Although there is no clear evidence to support a correlation between specific *PLA2G6* mutations and phenotype, it has been suggested that more aggressive forms of INAD are associated with nonsense and/or frameshift mutations while atypical NAD is more frequently found in patients with missense mutations^33^. Interestingly, it has been shown *in vitro* that mutations associated with INAD cause loss of enzyme activity. However, adult-onset dystonia-parkinsonism mutations do not impair PLA2G6 catalytic activity but may modify substrate preferences or its regulatory mechanisms^34^. Others have shown that a reduction in catalytic activity by about 70% is associated with homozygous *PLA2G6* mutation and adult-onset of PLAN^35^. Thus, these findings clearly indicate that we are still far from understanding how mutations in *PLA2G6* give rise to different phenotypes and clinical outcomes.

To gain insight into the mechanisms underlying PLA2G6-associated neurodegeneration, we developed a *Drosophila* model of calcium-independent phospholipase A2-VIA deficiency. We found that loss of iPLA2-VIA gave rise to age-dependent defects in climbing ability and spontaneous locomotion. Moreover, using a newly developed assay, we showed that *iPLA2-VIA* mutants also display impairments in fine-tune motor movements, motor coordination and psychomotor learning. Finally, we show that *iPLA2- VIA* mutants exhibit increased sensitivity to oxidative stress, progressive neurodegeneration and a severely reduced lifespan. These data are consistent with recent findings from Kinghorn *et al*.^26^ who also reported widespread vacuolation and mitochondrial dysfunction in the brain of *iPLA2-VIA* mutant flies.

The analysis of lifespan in *Drosophila iPLA2-VIA* mutants revealed several important findings. First, both *iPLA2-VIA* hypomorphic and null mutants exhibit severely reduced lifespans compared to genetic controls and display a rapid decline in survival during middle- age (~4 weeks old). This pattern is fully recapitulated by RNAi knockdown of *iPLA2-VIA* although the phenotype associated with ubiquitous knockdown resembles the null mutant phenotype while pan-neuronal knockdown is less severe and coincides with the lifespan deficits observed in the hypomorphic, P-element mutant. This data suggests that decreased lifespan correlates with decreased levels of *iPLA2-VIA* expression. Alternatively, the difference in lifespan between pan-neuronal and ubiquitous knockdown of *iPLA2-VIA* could suggest an essential requirement for iPLA2-VIA activity in other cells or tissues. Finally, both rescue and overexpression experiments demonstrated “stair-stepped” curves, with periods of prolonged survivorship. This suggests that the requirement for iPLA2-VIA may be age-specific. Taken together, these data suggest that lifespan can be regulated by a complex functional interplay between the levels of *iPLA2-VIA* expression, its spatial and temporal distribution and the age of the organism. Whether this accounts for the variable phenotypes associated with human PLAG26-neurodegeneration remains to be determined.

We next investigated whether *iPLA2-VIA* mutant flies displayed behavioral abnormalities related to symptoms of PLAN, such as impairments in motor functions, difficulties in climbing ability, maintenance balance and coordination. Based on data derived from longevity experiments we performed behavioral analyses in mutant and control flies at the same chronological and physiological age so that we could distinguish age-related defects from disease-related changes. A negative geotaxis assay revealed significant impairments in climbing ability in four-week old mutant flies compared to chronological and physiological controls. A detailed analysis of several aspects of locomotor behavior confirmed these findings. Mutant *iPLA2-VIA* flies spent significantly less time in locomotor activity and covered a shorter distance in a 10-min period. They also displayed a reduction in walking speed although this defect was not as dramatic as the previous two parameters. Taking together these results imply that the locomotor defects observed in *iPLA2-VIA* mutants cannot simply be attributed to an inability to walk but may reflect more subtle impairments in fine-tune movements, balance, motor coordination and speed of movement. Indeed, several studies have shown that flies demonstrate immediate adaptations in body posture, leg kinematics and inter-leg coordination after removal of a hind leg thereby maintaining their ability to walk^36^. Moreover, they can compensate for small defects in locomotion by the presence of six legs and flexible inter leg coordination. In addition, blocking proprioceptive feedback inactivates sensory neurons in the fly’s legs and results in deficient step precision, however, interleg coordination and the ability to execute a tripod gait are unaffected^37^. Therefore, we further examined the possible effect of *iPLA2-VIA* mutation on psychomotor behavior using a recently developed method^27^. Psychomotor activity is defined as the fine relationships between sensory perception, cognition and movement^36^. Psychomotor abilities and learning underlie the performance of many fundamental human motor activities such as movement, coordination, manipulation, strength and speed as well as fine motor skills such as use of precision instruments or tools. Psychomotor retardation is recognized as one of the main clinical symptoms of PLA2G6-associated neurodegeneration^17^. Patients affected by PLAN often show a loss of previously acquired motor skills, difficulties and/or slowness in movement, impairments in motor planning and the organization of movement, gait instability, rigidity and impaired postural response. Our “equilibrist test” allowed us to clearly demonstrate that *iPLA2-VIA* mutant flies develop age-dependent psychomotor impairments and were unable to improve their motor skills as early as two-three weeks of age.

Cognitive decline is a typical and prominent feature in the majority of PLAN cases^38–41^. However, in our experiments, we were unable to detect any significant effect of *iPLA2-VIA* mutations on learning or short-term memory performance. Young 2–3 day old mutant flies were indistinguishable from controls in initial learning scores and exhibited similar memory decay rates 30-min after training. They also did not show significant differences from controls at two weeks of age. The failure to detect learning and memory impairments in *iPLA2-VIA* mutant flies can be explained by two possible reasons: wrong choice of learning task and/or inappropriate testing age. Indeed, several of the available reports indicate that patients with PLA2G6-associated neurodegeneration suffer from decline in working^42^ or visual^18^ memory performance. In rats, inhibition of *iPLA2-VIA* in the hippocampal- prefrontal cortex causes defects in spatial working memory^43^ or long-term memory retrieval^44^ that are not required for olfactory associative learning. Another possible explanation for the lack of the cognitive defects is that mutant flies at two weeks were too young to demonstrate age-dependent learning and memory impairments. In fact, two-week old *iPLA2- VIA* mutant flies did not show any obvious phenotypes in behavior or neurodegeneration. Unfortunately, the presence of a very strong locomotor deficit in our four-week old mutant flies prevents us from testing learning and memory at this age.

Several lines of evidence suggest that the observed age-dependent neurodegeneration in the *iPLA2-VIA* mutant flies is caused by lack of iPLA2-VIA activity and seems likely to be related to disease pathogenesis rather than to aging *per se*. First, numerous TUNEL-positive cells were found in the brains of both four-week old P element and null mutant flies while the brains of their chronologically and physiologically-matched controls were normal. Second, gene silencing of *iPLA2-VIA* in elav-expressing cells led to the same phenotype.

Interestingly, TUNEL-positive cells were found most prominently in the area of the optic lobes in both *iPLA2-VIA* mutants and knockdown flies. These observations are consistent with recent studies showing abnormal retinal and grossly abnormal ommatidial structure in aged *iPLA2-VIA* P-element mutants^26^.

Polyunsaturated fatty acids (PUFAs) attached to the sn-2 position of phospholipids are the major targets for free radical attacks that mediated membrane damage by lipid peroxidation. These peroxidazed PUFAs are removed from the membrane by PLA2 enzymes. Thus, loss of normal PLA2 activity or high-level accumulation of ROS that exceeds the capacity of the PLA2 repair system might lead to the mitochondrial dysfunction and ultimately result in cell death. The analysis of our oxidative stress resistance experiments has revealed several intriguing findings: (1) Young flies for both *iPLA2-VIA* mutant lines exhibit reduced resistance to H_2_O_2_ exposure. In particular, a strong negative effect is seen in the null mutants, which display almost a two-fold decrease in the resistance and, (2) *iPLA2-VIA* knockdown in Elav-specific cells has no effect on resistance to oxidative stress in young flies. It is unlikely that the absence of a deleterious effect of oxidative stress on survival of young *elav*-*GAL4* knockdown flies is due to the fact that the expression of *iPLA2-VIA* was not completely abolished since elav-*GAL4* knockdown had a highly significant negative effect on oxidative stress resistance in 28 day old flies. Secondly, knockdown using the same *elav-GAL4* driven RNAi line significantly shortened lifespan. At the same time, overexpression of *iPLA2-VIA* in elav-expressing cells enhanced oxidative resistance in both 7 and 42 day old flies. All together, these results support an important role of *iPLA2-VIA* in regulating the oxidative stress response, however the precise mechanisms remain to be elucidated.

As we have shown above the P-element insertional line is a strong hypomorphic *iPLA2- VIA* allele, with 30% expression level of wild type, while the new null mutant is a structural (deletion) and functional null. Although our null mutant exhibits similar behavioral and neurodegenerative phenotypes as the P-element line, null mutant flies display more severe phenotypes in longevity and H_2_O_2_ exposure. Both longevity and survival under oxidative stress represent integrative characteristics that are required either directly or indirectly for almost all cellular processes. These differences may become extremely relevant when assessing the ability of wildtype and human disease associated variants to rescue *iPLA2-VIA* mutants and to determine how each variant gives rise to distinct pathogenesis in PLAN diseases.

## Conclusions

Mutations in *PLA2G6* are associated with a variety of autosomal recessive neurodegenerative disorders. At present, the cellular mechanisms underlying PLA2G6 associated neuropathology are poorly understood. We have developed a *Drosophila* model that recapitulates many of the features observed in humans, including age-dependent defects in psychomotor learning, progressive neurodegeneration and a severely reduced lifespan.

*Drosophila iPLA2-VIA* mutants may help to shed light on the mechanisms underlying PLA2G6 disorders and can be used to identify novel modifiers and for drug discovery. Since many of the pathological hallmarks of PLA2G6 disorders including the accumulation of brain iron, are also observed in Alzheimer’s and Parkinsons disease, any genes or compounds that suppress these phenotypes may also have therapeutic effects in a broad spectrum of neurodegenerative diseases.

## Methods

### Flies stocks and maintenance

All stocks were raised on standard fly food, with a 12/12-h light/dark cycle, at 24 ± 1 °C and 45–50% relative humidity. The *Canton S* (*CS*) line was used as the wild-type control. The *w*^1118^ background control line was previously outcrossed for 10 generations with *Canton S*. Other lines including balancers, GAL4 and UAS lines were outcrossed for at least 5 generations with the Cantonized *w*^1118^ line. The fly stock *y*^1^ *w*^67c23^; P w[+mC] y[+mDint2] = EPgy2-*iPLA2-VIA*^EY05103^ (#15947) was obtained from the Bloomington Stock Center. Null mutant fly lines *iPLA2-VIA*^25FRT^ and *iPLA2*-*VIA*^28FRT^ were generated by FLP-mediated recombination between a pair of FRT sites. Fly stocks that carried FRT containing transposons were obtained from the Exelixis Collection at Harvard medical School: *w*^1118^; PBac-WH [f03063] and *w*^1118^; PBac-RB [e00344]. The stock P-hsFLP22, *y*^1^ w*; P- neoFRT82B bon21B/TM3, *Sb* (Bloomington Stock Center (#43660) was used as a source of FLP recombinase. The FLP-FRT-based method to obtain deletions was used essentially as described by^45^. Briefly, female flies that contained the PBac-WH [f03063] P-element were crossed to males that contained both P-hsFLP22 and PBac-RB [e00344]. To activate FLP expression, larvae were heat-shocked at 37 °C for 1hr for 4–5 consecutive days. The female progeny carrying a possible recombinant chromosome and thus containing a deletion as well as a chimeric transposon that contains residual of WH P-element with UAS sequence and a part of RB P-element with w+ marker were crossed to a male of a Balancer Stock for Chromosome 3. Then a series of diagnostic PCRs were performed to confirm the presence of a deletion. UAS-RNAi-*iPLA2-VIA* was obtained from Bloomington Stock Center (#36129, no off target gene). Dicer (Chromosome II) was introduced to this RNAi line to promote more efficient knockdown. All driver-GAL4 control lines that were used for knock-down experiments also contained UAS-Dicer. The UAS-iPLA2-VIA-V5 stock was generated by cloning the iPLA2-VIA cDNA into a pUAST vector containing a V5 Tag. The *iPLA2-VIA* cDNA was PCR amplified from RE23733 cDNA-clone from DGRC (*Drosophila* Genomic Resource Center, Bloomington, IN) using primers iPLA2-VIA-F – CGG CTC GAG GAG TCA TGG CGT GGA TGG CG and iPLA2-VIA-R – CCG CTC GAG TTT TAG GAA ATT GAT CAT CTC GAT. Transgenic lines were produced by BestGene Inc. To drive expression of the transgene the following GAL4 lines were used: *actin-5C*-*GAL4*, *daughterless*-*GAL4* and *elav*^c155^*-GAL4* lines (Bloomington Stock Center).

### Molecular biology and transgenics

#### Western blot

For Western blots 7-day-old flies were collected on ice and homogenized in cold RIPA buffer with protease inhibitors. 100 ug of protein extract of each genotype was loaded per a well of a 10% Polyacrylamide gel. Proteins were run in SDS buffer and transferred to PVDF membrane and then blocked: 5% milk, 5% BCA, 4%FBS, 2%NGS in TBST overnight at +4 °C. To detect V5-Tag the membrane was incubated for 1 h in 1% milk, 1%BSA, 1%FBS, 1%NGS in TBST with V5 Epitope Tag Antibody (1:5000; Thermo Fisher, R960-25) at RT. After washes and incubation with anti-mouse HRP-coupled secondary antibodies (1:10000; Jackson ImmunoResearch) ESL reagent was used to detect the HRP signal. The membrane was stripped using stripping solution (2%SDS, 120 mM Tris-HCl pH6.8, 0.7% 2- Mercaptoethanol) and incubated with E7 β-tubulin primary antibodies (1:1000; Developmental Studies Hybridoma Bank), which was used as a loading control, followed by washes and 1 h RT incubation with secondary HRP-coupled antibodies.

#### RT-PCR and qRT-PCR

For Reverse Transcriptase PCR (RT-PCR) total RNA was isolated from at least 15 adult flies (about 1 week old) and treated with DNase I using High Pure RNA Isolation Kit (Roche, Ref 11 665 828 001). cDNA synthesis was performed using SensiFAST cDNA Synthesis Kit (BioLine, BIO-65054). PCR was set up with cDNA as a template and two pairs of primers specific for iPLA2-VIA genomic sequence that carried an intron to distinguish any non-specific PCR product than can arise from remaining genomic DNA: 1(001 R) GGT GTC GAT CAG CCG GCC AAT AAG G and 2(003 L) GAT TAT CAG CCA AGG CAT CGC AAG; 3(0136 R) AAC CCG ACT CTA GAC GCC ATG AC and 5(033 L) GTA GGG TAT GCC AAT CGT GCT GC. Loading control was set up with primers specific to the sequence of rp49: fwd AGT GCG TCG CCG CTT CAA GG; rev AGA ACG CAG GCG ACC GTT GG. For quantitative real-time RT-PCR (qRT-PCR) total RNA was isolated from the whole body of 7–9-day-old adult flies using the TRIzol method (Invitrogen) according to the manufacturer’s instructions. Genomic DNA was removed by 15 min RNase-free DNase I (Invitrogen) digestion. mRNA from total RNA was reverse transcribed to cDNA using oligo(dT) primer and the SuperScript II system (Invitrogen).

qRT-PCR was carried out on the LightCycler 480 Real-Time PCR machine using the SYBR green detection (Roche). Each qRT-PCR was performed using three independent biological replicates, each sample was assayed in triplicates, and at least 15 flies were taken per sample. qRT-PCR results were normalized to an internal control (rp49). The following primers were used for qPCR: iPLA2-VIA: 3(016 R) AAC CCG ACT CTA GAC GCC ATG AC; 4(017 L) CAG TTT GGC CGT ATC CCA GAT GC.

#### qRT-PCR of Knockdown and Overexpression lines

Total RNA was isolated from at least 15 adult flies (about 1 week old) or 50 heads per sample using High Pure RNA Isolation Kit (Roche, Ref 11 665 828 001). cDNA synthesis was performed using SensiFAST cDNA Synthesis Kit (BioLine, BIO-65054). For quantitative RT-PCR (qRT-PCR) specific probes were used for iPLA2-VIA (TaqMan Probe, Life Technologies, Assay ID Dm01801945_g1) and for the internal control rp49 (Assay ID 02151827_g1) along with SensiFAST Probe Lo-ROX One Step Kit (BioLine; BIO-78001). Reactions were carried out in a qRT-PCR machine ViiA7 (Applied Biosystems). Each qRT- PCR was performed using three independent biological replicates, each sample was assayed in triplicate. qRT-PCR results were normalized to an internal control (Rp49).

### Lifespan

All experimental flies were collected within 6 h after eclosion, and sorted into mixed groups consisting of 20 flies (10 males and 10 females) per vial. Flies were transferred to fresh food vials almost every second day. Survivorship was scored at the time of transfer until all flies died. All vials were maintained with foam stoppers and placed on their side to prevent flies from getting stuck in the medium. Statistica software package, version 13.0 (Statsoft. Inc., Tulsa, USA) was used to generate the Kaplan–Meier survivorship curves that were compared using Mantel–Cox log-rank tests for estimation of the life-span values. Experiments were done in a special environment room, with a 12/12-h light/dark cycle, at 24 ± 1 °C and 60% relative humidity.

### Oxidative stress

For oxidative stress assays males were first placed on 1% agar vials (20 males/vial) for 2 h starvation and then transferred to vials with filters (Life Sciences, Whatman; N 1001-020) that were soaked in 5% sucrose solution containing 2% hydrogen peroxide (Sigma, 216763). To keep filters moist all the time during each experiment the appropriate solutions were delivered to filters once in a 24 hour period.

### Locomotion and psychomotor behavior

#### Climbing behavior

Climbing behavior of adult flies was measured using a countercurrent apparatus essentially as described^46^. Briefly, groups of approximately 50 flies (both males and females) were given 60 sec to adapt in the starting tube, which can slide along the apparatus and then 25 seconds to move upwards against gravity to the upper frame’s tube. The top frame of tubes then was shifted to the right so that start tube comes into register with second bottom tube and flies, which successfully climbed up were tapped down again, falling into tube 2. The upper frame is returned to the left and flies are once again allowed to climb into the upper tube. After five runs, the number of the flies in each tube was counted. For each time point, at least eight cohorts from each genotype were scored. The Climbing Index (CI) was calculated using the following formula: CI (the weighted mean) = Σ(*mn*_*m*_)/N. CI is ranged from 1 (min) to 6 (max). Here: *m* – number of test vial, *nm* – amount of flies in the *m*^*th*^ vial, N – total amount of flies.

#### Spontaneous locomotor activity, psychomotor activity and learning, Video-Tracking and Data Analysis

All behavioral experiments were performed with wing-clipped males within a 3-hour time window (between 16.00–19.00 h) in an environmental control room. The wings of males were clipped at least three days before experiments were performed under light CO_2_ anesthesia. To test spontaneous locomotor activity we used custom made 6-well chambers (35 mm diameter and 7 mm high). Individual flies were gently aspirated into the chambers 30 min prior to recording activity to allow adaptation to the new environment. A color camera (EverFocus EQ. 610, Polistar II) was fitted with a CCTV lens (Computar, Vari Focal TG4Z2813 FCS-IR) and fixed on a mounting bracket about 50 cm above the chambers. The distance of the camera to the object as well as the zoom, focus and iris aperture were optimized for video-tracking. The path of freely moving flies within the arena was tracked during 10 min. with Ethovioson XT v.10 (Noldus Information Technology, Leesburg, VA). The setup for testing psychomotor activity and learning consists of two platforms submerged within a pool, with only the small upper parts 5 mm over the water surface. A clear fishing line (0.6 mm) was then strung between two platforms. A single fly was then gently removed from a vial by aspirator and placed on the platform. Video-tracking was initiated when the fly entered a zone (colored in red), which is 1 cm away from both platforms. The learning protocol consisted of three sessions, each separated by one day. Each session consists of three successive trials (without intervals in between). More details about this assay see^27^.

The trajectories obtained from video-tracking were analyzed using the following parameters: the total distance covered in 10 minutes, percent of time spent in locomotion, the walking speed in open filed tests and the walking speed in the “equilibrist test”. To reduce any effects due to nonspecific jerky movements and/or wobbling we defined a fly to be moving when it had traveled a minimum of 4 mm/s and a fly to be stopped when its speed was 2 mm/s or less during locomotor activity. For the “equilibrist test” we defined a fly to be moving when its speed was 2 mm/s or higher. These parameters were averaged for five data points as a sliding window over the total recording time^47^.

### Neurodegeneration

For TUNEL analysis, brains from different age group flies were dissected in cold PBS, fixed in 4% PFA for 25 min at room temperature. Brains were rinsed twice in PBS for 5 min and washed once in H_2_O plus 0.1% Triton X-100 and 0.1% Sodium citrate for 15 min at room temperature and then twice in PBS. TUNEL staining was performed by following the manufacture’s instruction (Roche, *In situ* cell death detection kit, Cat # 11684795910). Images were captured as a Z-stack using a Leica SP8 confocal microscope and Z-stacks were compressed into a single image.

### Pavlovian Olfactory Conditioning

Learning and testing procedures as well as sensorimotor responses were essentially done as described by Krashes and Waddell^48^ with some minor changes. Briefly, to induce aversive associative olfactory memory, groups of about 75 flies were exposed for 60 s sequentially to two odors. The first odor was paired with an electric shock (60 V), while the second was not. During the test flies were exposed to both odors simultaneously in a T-maze for one minute. The performance index (PI) was calculated as the percentage of flies avoiding the odor paired with electric shock minus the percentage of flies avoiding the unpaired odor. The paired and unpaired odors were swapped in the two reciprocal halves of each experiment.

The final PI was the average of the two reciprocal PI values. As odorants, 22.5 μL or 150 μL 3-Octanol (Oct), 15 μL or 150 μL 4-Methylcyclohexanol (MCH) were added to the vial with 15 mL of mineral oil and vortexed. All training and testing procedures were performed in a climate controlled room with 70–75% humidity at 25 °C under dim red light.

### Statistical procedures

All data were tested for normality by the Kolmogorov-Smirnov test. Non-normally distributed data was log transformed before calculation of means and errors. One-way ANOVA or Student’s *t* test was applied when variances were homogeneous. In cases of non- equal variances, an approximate *t* test was used. For analysis of lifespan and oxidative resistance data the Statistica software package was used to generate the Kaplan–Meier survivorship curves that were compared using Mantel–Cox log-rank tests and for estimation of life-span values. For analysis of locomotor activity, the behavioral data were Box–Cox transformed to correct problems of distribution and non-homogeneity of variance before transformation. Normal distribution was confirmed by the Kolmogorov-Smirnov test. Two- way analysis of variance and subsequent Tukey HSD post hoc comparisons were performed to evaluate differences among genotypes and age. Statistical analyses were conducted using Statistica 13.0 (Dell Inc. (2015) and Dell Statistica (data analysis software system) software. A general linear model (GLM) was constructed to estimate the association between performance in “equilibrist test” (mean of the walking speed) and number of trials. Post-hoc analysis was conducted using Tukey HSD method.

### Data availability

The datasets generated during and/or analysed during the current study are available from the corresponding author on reasonable request.

## Electronic supplementary material

Supplementary material
