# Supplementary material for: Mutations in the Drosophila homolog of human PLA2G6 give rise to age-dependent loss of psychomotor activity and neurodegeneration
Source: Sci Rep. 2018 Feb 13;8:2939. doi: 10.1038/s41598-018-21343-8 (PMC5811537; doi:10.1038/s41598-018-21343-8)

Supplementary material: Mutations in the *Drosophila* homolog of human PLA2G6 give rise to age-dependent loss of psychomotor activity and neurodegeneration

Konstantin G. Iliadi, Oxana B. Gluscencova, Natalia Iliadi and Gabrielle L. Boulianne

[1]Program in Developmental and Stem Cell Biology, The Hospital for Sick Children, Toronto, Ontario M5G 1L7, Canada, and [2]Department of Molecular Genetics, University of Toronto, Toronto, Ontario M5S 1A8, Canada

Supplemental Figure S1: Full-length gel images of the RT-PCR analysis of iPLA2-VIA alleles showing absence of expression in the null iPLA2-VIA25FRT mutant and presence of the RT-PCR product in the iPLA2-VIAEY05103.

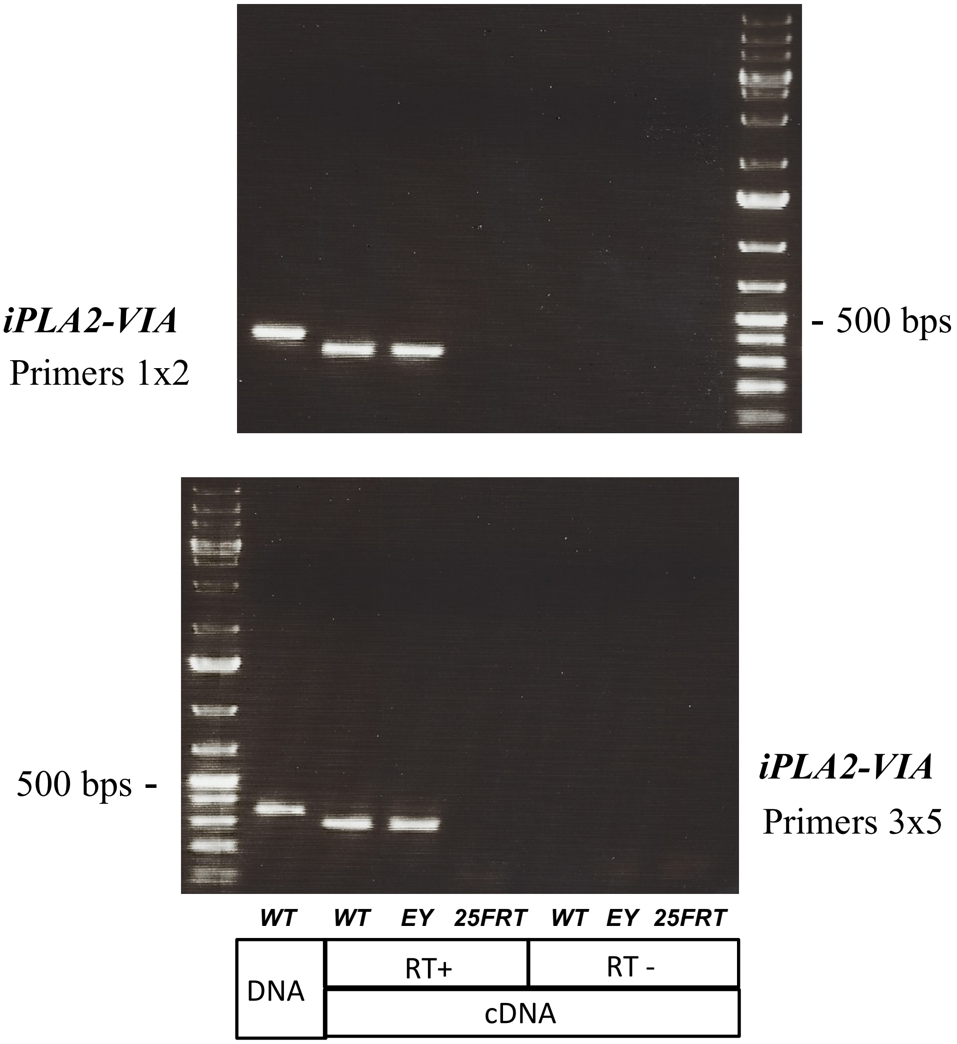

Supplemental Figure S2. Rp49 loading control for the RT-PCR of iPLA2-VIA shown on figure S1.

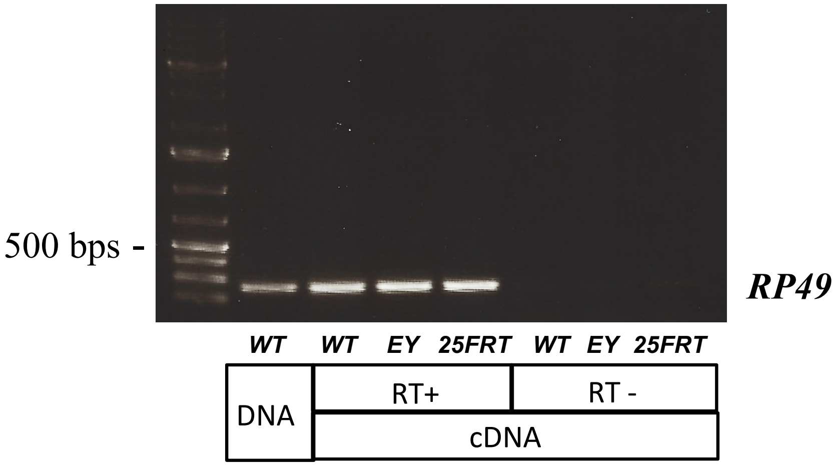

Supplemental Figure S3. Full-length gel images of the western blot probed with (upper blot) anti-V5 tag shows expression of different UAS-iPLA2-VIA lines and tubulin loading control (lower blot).

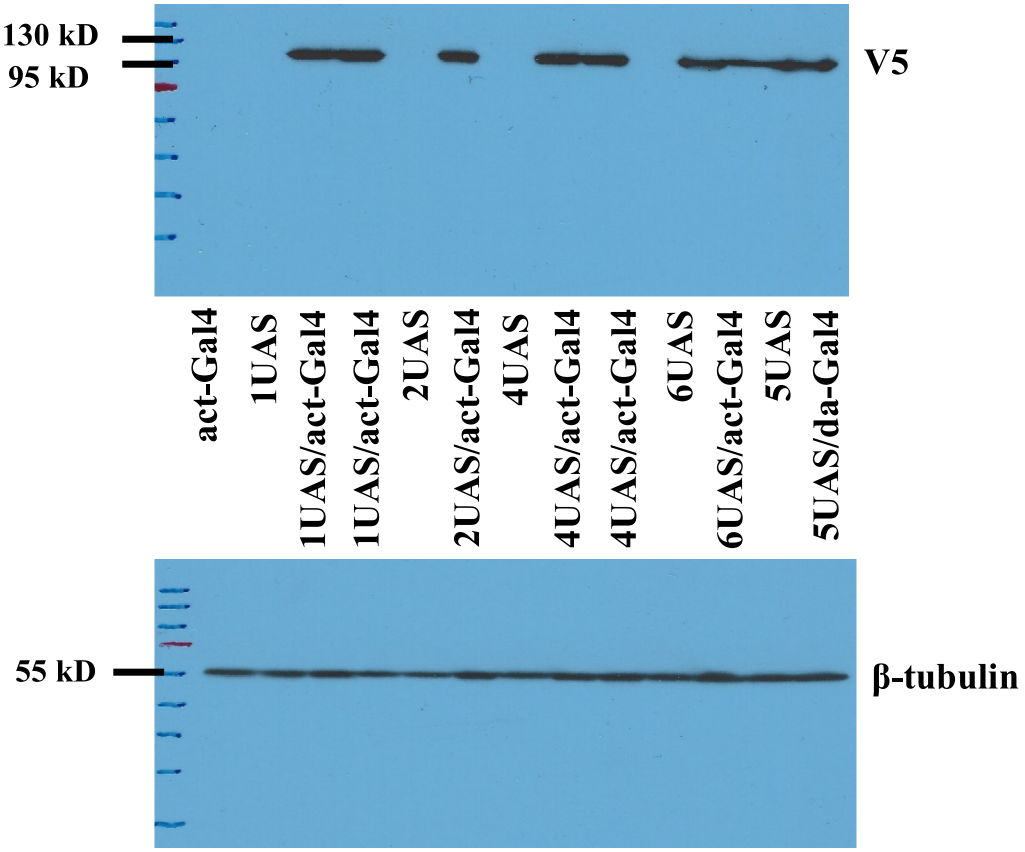

Supplement: Supplementary file 1 — Supplementary material [file 41598_2018_21343_MOESM1_ESM.pdf]
